# The Changing Landscape of Molecular Diagnostic Testing: Implications for Academic Medical Centers

**DOI:** 10.3390/jpm6010008

**Published:** 2016-01-27

**Authors:** Heidi L. Rehm, Elizabeth Hynes, Birgit H. Funke

**Affiliations:** 1 Laboratory for Molecular Medicine, Partners HealthCare Personalized Medicine, Boston, MA 02139, USA; hrehm@partners.org (H.L.R.); eduffy1@mgh.harvard.edu (E.H.); 2 Department of Pathology, Brigham and Women’s Hospital and Harvard Medical School, Boston, MA 02115, USA; 3 Department of Pathology, Massachusetts General Hospital and Harvard Medical School, Boston, MA 02114, USA

**Keywords:** molecular diagnostics, next generation sequencing, academic medical centers, Laboratory of Molecular Medicine, Partners HealthCare, comprehensive testing, laboratory–physician collaborations, clinical translational research, biobank, commercial–academic partnerships

## Abstract

Over the last decade, the field of molecular diagnostics has undergone tremendous transformation, catalyzed by the clinical implementation of next generation sequencing (NGS). As technical capabilities are enhanced and current limitations are addressed, NGS is increasingly capable of detecting most variant types and will therefore continue to consolidate and simplify diagnostic testing. It is likely that genome sequencing will eventually serve as a universal first line test for disorders with a suspected genetic origin. Academic Medical Centers (AMCs), which have been at the forefront of this paradigm shift are now presented with challenges to keep up with increasing technical, bioinformatic and interpretive complexity of NGS-based tests in a highly competitive market. Additional complexity may arise from altered regulatory oversight, also triggered by the unprecedented scope of NGS-based testing, which requires new approaches. However, these challenges are balanced by unique opportunities, particularly at the interface between clinical and research operations, where AMCs can capitalize on access to cutting edge research environments and establish collaborations to facilitate rapid diagnostic innovation. This article reviews present and future challenges and opportunities for AMC associated molecular diagnostic laboratories from the perspective of the Partners HealthCare Laboratory for Molecular Medicine (LMM).

## 1. Introduction

Molecular diagnostic testing has undergone dramatic changes since its debut in the late twentieth century. Initially restricted to simple assays interrogating single to a few genomic sites known to harbor pathogenic variants, the adoption of Sanger sequencing enabled whole gene analysis and therefore the discovery of rare and novel disease causing variants. Over the last decade, the field has undergone another tremendous transformation, catalyzed by the clinical implementation of next generation sequencing (NGS). This disruptive technology enabled replacement of single gene tests and small gene panels with large, comprehensive gene panels and more recently whole exome and genome sequencing, reducing the need for lengthy and expensive stepwise testing algorithms and therefore eliminating many diagnostic odysseys [1,2,3,4]. A similar evolution has already taken place in the cytogenomic community where genome wide copy number testing quickly replaced older approaches. 

In 2015, comprehensive NGS is the standard for most inherited disorders with genetic and clinical heterogeneity although limitations surrounding detection of structural and other complex variants often necessitate additional testing. This typically requires use of different technology platforms such as cytogenomic and exon arrays, which continue to be the methods of choice for detecting copy number variants (CNVs). Other variants that are non-trivial to detect by NGS include copy number neutral structural rearrangements (translocations, inversions) and repeat expansions. However, it is already evident that NGS will continue to catalyze consolidation of diagnostic testing approaches. For example, NGS-based CNV detection is being implemented in diagnostic laboratories [5,6,7,8] and has the potential to serve as a convenient “one stop shop” for laboratories. Likewise, innovative approaches to detect repeat expansions and other difficult to sequence variation are being developed. Overall, this path is likely to lead to a single whole genome analysis approach that could serve as a first line test for detecting all types of germline DNA variation. 

## 2. Implications for Academic Medical Center (AMC) Associated Diagnostic Laboratories

The rapidly growing scope of genomic testing has implications for many small to medium sized AMC associated diagnostic laboratories, including challenges but also opportunities. 

*Challenges:* New genomic platforms require significant capital investment and typically a high test volume to be able to operate at scale and achieve competitive price points. In addition, establishing and maintaining different technology platforms is difficult. The emerging practice of offering comprehensive sequence and copy number testing illustrates this challenge. Access to genome wide cytogenomic CNV detection platforms can be an advantage until NGS is equally accurate. However, cytogenomic and molecular laboratories have historically been separately managed at AMCs, which can be an obstacle towards integrating these technology platforms to streamline comprehensive testing. Furthermore, the necessary capital to establish the infrastructure for modern genomic testing and comprehensive interpretation is often not present at AMCs, especially in the current funding climate. For example, automation is increasingly important for supporting complex genomic testing processes but requires a test volume that is often not present at AMCs. Commercial entities have an increasing presence in this space and are often better equipped to establish a cohesive suite of technology platforms to meet all testing needs and maintain a higher throughput and therefore lower price points. 

A second major challenge is the recruitment and retention of staff needed for high complexity genomic testing. The level and diversity of skill sets needed has grown significantly, making it difficult to recruit highly trained staff within the salary constraints present at academic institutions. This is especially problematic in two areas that have rapidly increased as large scale genomic tests become the norm: (1) The associated need for computational (bioinformatics and IT) support has proven to be extremely difficult to manage for diagnostic laboratories in several ways. First, computational/bioinformatic skills are typically present in service oriented core facilities, which serve researchers in a project oriented fashion. Diagnostic laboratories are subject to regulatory oversight and therefore the traditional computational support model does not meet all needs, which range from project-like efforts such as the development of novel genetic tests to all aspects of routine operations including quality management. As such, computational analysts and bioinformaticians need to be integrated into clinical operations and need to be trained in genetics as well as regulatory requirements, as regulators are extending oversight to computational processes; (2) Traditional (pre-Sanger) diagnostic testing required a mix of technical and interpretive work that was skewed towards technical analytic management. With the ability to interrogate large numbers of genes, this balance has been shifted significantly towards clinical interpretation of technical results, which has created a tremendous overhead associated with curating and managing genomic knowledge. The end result is an increased need for highly trained (and therefore expensive) MD or PhD level staff. While AMCs have easy access to such staff, it can be difficult to secure a sufficient level of funding to capture this resource.

In addition, molecular diagnostic testing is currently under intense scrutiny as the US Food and Drug Administration (FDA) has announced its intention to begin regulating laboratory developed tests (LDTs) after decades of using “enforcement discretion”. This shift was in part catalyzed by the rapid adoption of NGS-based tests in clinical testing laboratories and the realization that traditional regulatory frameworks, which were largely designed for genotyping tests that interrogate known, pathogenic variants, are completely inappropriate to establish and monitor the analytic validity of assays that sequence a large number of genes in their entirety and therefore detect novel variation. In 2015, there is no clarity on how the US regulatory framework will evolve. Although any changes would affect commercial entities and AMCs alike, the latter are generally ill equipped to meet the significant financial overhead associated with traditional FDA approval processes. This has created an immense and justified fear of stifling innovation, which often originates at AMCs. Finding the right balance between regulation and flexibility to ensure quality as well as enable rapid translation of novel findings and technological advances is critical, especially in an era where we are routinely seeing the benefits of genetic and genomic testing.

*Opportunities:* AMC associated diagnostic laboratories can often achieve a tight integration with local treating physicians, which benefits both specialties. Ideally, laboratory directors and physicians collaborate to form “physician–laboratory partnerships”, which are of growing importance as genomic testing is increasingly utilized as one of several modalities to establish a clinical diagnosis. Board certified PhD or MD level laboratory directors are broadly trained in clinical and technical disciplines but rarely reach the knowledge of a clinical expert. Such partnerships can therefore provide a critical foundation for maximizing the clinical utility of tests, especially in clinical areas that are new for a testing laboratory. Laboratory–physician collaborations include operational activities such as selecting the most relevant genes that need to be interrogated for a particular disorder. Clinical domain knowledge is also helpful to determine whether additional, supplemental assays are required to ensure complete and clinically meaningful interrogation of a gene. In addition, laboratory directors benefit from a solid clinical knowledge base, which enhances the clinical interpretation of test results. Another opportunity involves the clinical translational research environment common to AMC associated diagnostic laboratories. Early leadership in translational programs affords AMC associated diagnostic labs an innate ability to innovate, allowing new discoveries to be rapidly translated into clinical tests and to support clinical trials where robust patient recruitment and close interaction with the clinical care environment are often necessary. This integration with clinical translational research is especially potent at those AMCs operating large consented biorepositories, which are poised to accelerate genomic discoveries with diagnostic relevance.

## 3. Portrait of a Medium Sized AMC Associated Molecular Diagnostic Laboratory

**Figure 1:**
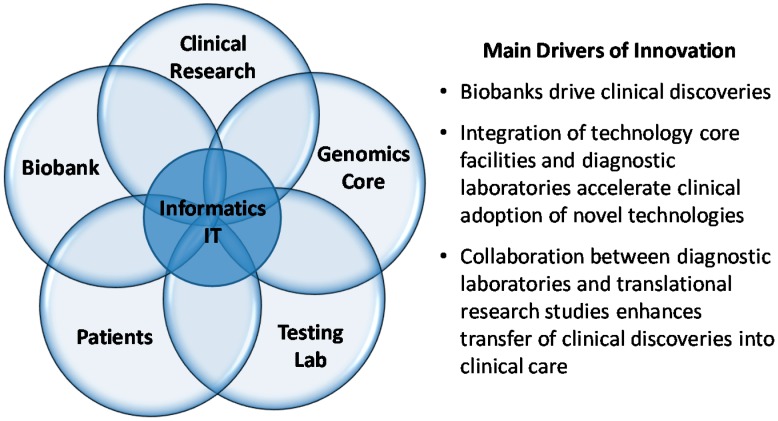
Integrated framework to enable rapid bench to bedside translation at Academic Medical Centers (AMCs).

The Laboratory for Molecular Medicine (LMM) was founded 13 years ago as part of a broader program, now called Partners Healthcare Personalized Medicine that also includes translational genomics core service, bioinformatics and IT services, and a biobank (http://personalizedmedicine.partners.org/). The LMM’s mission is to bridge the gap between research and clinical medicine by accelerating the adoption of new molecular tests into clinical care. An integral component of this mission is the incorporation of advanced computational and IT support into the day to day operations of the clinical laboratory, as well as implementing innovative programs to help physicians stay current on genetic information relevant to their patients. The LMM offers disease-targeted NGS panels for a variety of disorders with genetic and clinical heterogeneity (covering ~400 genes) as well as exome and genome sequencing services with an average of ~5000 high complexity genetic and genomic tests/year. Major areas of expertise include inherited cardiomyopathies, hearing loss, respiratory disorders, connective tissue disorders, RASopathies, and multi-organ genetic syndromes. LMM’s ability to innovate quickly is due to its strong academic focus (all directors have faculty appointments at local AMCs and are actively involved in clinical research), its close interaction with local physicians and its proximity and collaboration with the Center’s translational genomics research core as well as its bioinformatics team. The linkage to core facilities supporting research has enabled rapid transfer of novel technologies into the clinic, most notably enabling the LMM to be an "early adopter" of clinical NGS [9] and assume a national and international leadership role in this space. [10,11,12] (Figure 1). 

Key distinguishing features enabling the realization of LMM's mission include management of key clinical testing areas by small disease focused teams of 1–2 genetic counselors and fellows under the leadership of an American Board of Medical Genetics and Genomics (ABMGG) certified director and a tight, “matrixed” integration of its compartments (clinical test operation, clinical test development) and linkage with other Center cores. For example, most LMM directors have leadership roles either in other parts of the clinical operation (test development) or other parts of the Center (translational genomics core and bioinformatics). By linking these typically separate components through ABMGG certified individuals, many of the challenges mentioned above can be alleviated and synergy is created that benefits not only the clinical laboratory but also the other parts of the Center. Several examples illustrating the benefit of this infrastructure are described below. 

*Clinical test development*: Novel technologies typically make their debut in the research realm and are optimized over a period of time until they are mature enough for diagnostic implementation. The co-location of the LMM and the Center’s translational genomics core as well as the overlap in staff enabled clinical adoption of NGS, which was clinically launched at the LMM in 2011. Prior to NGS implementation, the LMM was the first laboratory to develop clinical grade oligo-hybridization-based sequencing which supported an interim platform for disorders with genetic heterogeneity starting in late 2007 [13,14,15]. *Clinical Research:* There is an increasing demand to carry out translational research studies in a CLIA environment. As this is associated with increased cost, one approach is to generate the initial data in a research environment and confirm and interpret actionable findings in the CLIA lab. This generates a revenue source for the CLIA lab and a competitive advantage for the research core. *Fluid team structures*. Strategic staffing approaches are of growing importance in maximizing efficiency, especially under financial constraints that are often present in AMC associated laboratories. Three examples illustrate this concept: (1) a critical activity of clinical diagnostic laboratories is quality improvement (QI), which requires expertise that is present in the clinical test development as well as operations teams. At the LMM, senior clinical operations staff participate in quality improvement projects as they operate and/or oversee the technologies on a day to day basis. Overall, QI is jointly managed by the clinical test development team in collaboration with the laboratory’s operational leadership by selecting and overseeing an ad hoc team composed of development and operations staff with expertise in the specific technology underlying the QI effort; (2) Translational Core staff often participate in clinical test development as the developed clinical tests are often offered by the Core as a research grade service; (3) Most parts of the clinical lab require substantial computational and bioinformatics support. On a day to day basis this typically entails analyzing large data sets and basic programming. The LMM development team is staffed by a computational analyst with strong training in molecular genetics and genomics technologies as well as a senior bench technologist with computational and basic programming skills. These individuals participate in regular meetings of the Center’s bioinformatics core, thereby embedding computational expertise directly within the clinical lab and facilitating closer collaborations when more substantial bioinformatics support is needed. This fluid interaction with the bioinformatics Core, as opposed to seeking support from a classic core facility, has proven to be crucial in efficient clinical test development.

## 4. Commercial–Academic Partnerships

Given the challenge of maintaining sufficient financial resources to sustain AMC-associated diagnostic laboratories, AMCs have increasingly turned to commercial partnerships to ensure financial stability. These may take the form of acquisitions or partnerships with outside commercial entities (e.g., Baylor-Miraca, Emory-Eurofins) or licensing of knowledge, software, or tests developed by an AMC-associated laboratories (e.g., My Cancer Genome-GenomOncology, GeneInsight-Sunquest). These relationships present both challenges and opportunities to the laboratories. Revenues streams and financially secure business partnerships enable AMCs to sustain their innovation and ability to evolve efficiently within an increasingly competitive market; however, conflicts of interest and commercial motivations can create barriers and shifted priorities that can be at odds with advancing the academic mission of an AMC. Setting clear boundaries and expectations as well as ensuring transparency in all relationships is necessary to achieve optimal outcomes in the rapidly advancing framework of genomic medicine. 

Over the past 10 years, the LMM has worked closely with the Center’s software development team building the GeneInsight platform [16]. While this robust and tight relationship led to the development of a highly innovative and well-developed software solution for genetic and genomic reporting and knowledge management, the high cost of maintaining the system and IT staff led to the decision to partner with Sunquest in 2015 to enable robust support for commercialization of the software. The transfer of staff support and software development costs to Sunquest has reduced the LMM’s overhead enabling revenue to be diverted to reducing tests costs and innovating in other areas such as knowledge management and new genomic test development.

## 5. Future Directions

As AMC-associated diagnostic laboratories look to the future, several options enable a sustained contribution to the genetic and genomic testing market. These include: (1) Partnerships with commercial entities to compete in the rapidly evolving global genetic and genomic testing market, (2) Focus on local testing needs including support for higher volume tests and/or conversion of all rare disease testing to a common genomics platform and (3) Shifting focus to support for clinical research programs and other project-oriented endeavors.

The LMM has been increasingly pursuing path #3 and shifted its focus to lead or support clinical research partnerships. Examples include supporting the NIH-funded MedSeq [17] and BabySeq projects, the Geisinger–Regeneron Partnership, and the eMERGEIII NHGRI consortium as well as building strategic partnerships with technology innovators such as the Broad Institute’s Clinical Research Sequencing Platform. Furthermore, we see an increasing and critical need to support the sharing of clinical genomic knowledge and data including the expansion of community-driven approaches to support the most effective use of genomics in clinical care as exemplified by the NIH-funded ClinGen project [18]. We encourage other AMCs to prioritize this mission and ensure the development of infrastructure and approaches that facilitate data-sharing and participation in community efforts to development rich and reliable knowledge sources free from commercial constraints. While laboratories compete in clinical service offerings, participation in data sharing and contribution to a common knowledge resource must be considered precompetitive to ensure that optimal patient care remains the highest priority in the practice of medicine.

## References

[1] Xue Y., Ankala A., Wilcox W.R., Hegde M.R. (2015). Solving the molecular diagnostic testing conundrum for Mendelian disorders in the era of next-generation sequencing: Single-gene, gene panel, or exome/genome sequencing. Genet. Med..

[2] Sawyer S.L., Hartley T., Dyment D.A., Beaulieu C.L., Schwartzentruber J., Smith A., Bedford H.M., Bernard G., Bernier F.P., Brais B. (2015). Utility of whole-exome sequencing for those near the end of the diagnostic odyssey: Time to address gaps in care. Clin. Genet..

[3] Richards J., Korgenski E.K., Srivastava R., Bonkowsky J.L. (2015). Costs of the diagnostic odyssey in children with inherited leukodystrophies. Neurology.

[4] Neveling K., Feenstra I., Gilissen C., Hoefsloot L.H., Kamsteeg E.J., Mensenkamp A.R., Rodenburg R.J., Yntema H.G., Spruijt L., Vermeer S. (2013). A post-hoc comparison of the utility of Sanger sequencing and exome sequencing for the diagnosis of heterogeneous diseases. Hum. Mutat..

[5] Pugh T., Amr S., Bowser M., Gowrisankar S., Hynes E., Mahanta L., Rehm H., Funke B., Lebo M. (2015). VisCap: Inference and visualization of germline copy number variants from targeted clinical sequencing data. Genet. Med..

[6] Retterer K., Scuffins J., Schmidt D., Lewis R., Pineda-Alvarez D., Stafford A., Schmidt L., Warren S., Gibellini F., Kondakova A. (2015). Assessing copy number from exome sequencing and exome array CGH based on CNV spectrum in a large clinical cohort. Genet. Med..

[7] Feng Y., Chen D., Wang G.L., Zhang V.W., Wong L.J. (2015). Improved molecular diagnosis by the detection of exonic deletions with target gene capture and deep sequencing. Genet. Med..

[8] Tattini L., D’Aurizio R., Magi A. (2015). Detection of genomic structural variants from next-generation sequencing data. Front Bioeng. Biotechnol..

[9] Gowrisankar S., Lerner-Ellis J.P., Cox S., White E.T., Manion M., LeVan K., Liu J., Farwell L.M., Iartchouk O., Rehm H.L. (2010). Evaluation of second-generation sequencing of 19 dilated cardiomyopathy genes for clinical applications. J. Mol. Diagn..

[10] Gargis A.S., Kalman L., Berry M.W., Bick D.P., Dimmock D.P., Hambuch T., Lu F., Lyon E., Voelkerding K.V., Zehnbauer B.A. (2012). Assuring the quality of next-generation sequencing in clinical laboratory practice. Nat. Biotechnol..

[11] Rehm H.L. (2013). Disease-targeted sequencing: A cornerstone in the clinic. Nat. Rev. Genet..

[12] Rehm H.L., Bale S.J., Bayrak-Toydemir P., Berg J.S., Brown K.K., Deignan J.L., Friez M.J., Funke B.H., Hegde M.R., Lyon E. (2013). ACMG clinical laboratory standards for next-generation sequencing. Genet. Med..

[13] Zimmerman R.S., Cox S., Lakdawala N.K., Cirino A., Mancini-DiNardo D., Clark E., Leon A., Duffy E., White E., Baxter S. (2010). A novel custom resequencing array for dilated cardiomyopathy. Genet. Med..

[14] Waldmüller S., Müller M., Rackebrandt K., Binner P., Poths S., Bonin M., Scheffold T. (2008). Array-based resequencing assay for mutations causing hypertrophic cardiomyopathy. Clin. Chem..

[15] Teekakirikul P., Cox S., Funke B., Rehm H.L. (2011). Targeted sequencing using Affymetrix CustomSeq Arrays. Curr. Protoc. Hum. Genet..

[16] Aronson S.J., Clark E.H., Babb L.J., Baxter S., Farwell L.M., Funke B.H., Hernandez A.L., Joshi V.A., Lyon E., Parthum A.R. (2011). The GeneInsight Suite: A platform to support laboratory and provider use of DNA-based genetic testing. Hum. Mutat..

[17] Vassy J.L., Lautenbach D.M., McLaughlin H.M., Kong S.W., Christensen K.D., Krier J., Kohane I.S., Feuerman L.Z., Blumenthal-Barby J., Roberts J.S. (2014). The MedSeq Project: A randomized trial of integrating whole genome sequencing into clinical medicine. Trials.

[18] Rehm H.L., Berg J.S., Brooks L.D., Bustamante C.D., Evans J.P., Landrum M.J., Ledbetter D.H., Maglott D.R., Martin C.L., Nussbaum R.L. (2015). ClinGen—The clinical genome resource. N. Engl. J. Med..

